# Integrating augmented reality technology into physical therapy in the school setting: a feasibility study

**DOI:** 10.1080/07853890.2025.2499022

**Published:** 2025-05-31

**Authors:** Elise Baron, Amy Pratt, Betsy Donahoe-Fillmore

**Affiliations:** ^a^ Augment Therapy^®^, Cleveland, OH, USA; ^b^ Montgomery County ESC, Dayton, OH, USA; ^c^ Department of Physical Therapy, University of Dayton, Dayton, OH, USA

**Keywords:** Physical therapy, augmented reality, students, patient participation, exergaming

## Abstract

**Purpose:**

To determine the feasibility of using an immersive augmented-reality technology (AT) in conjunction with physical therapy (PT) for in-school therapy, while determining whether this technology would be as engaging and motivating as standard PT.

**Methods:**

Participants included Fifteen students (6–8 y/o) receiving once-weekly PT. The therapist was instructed to engage students in AT and standard PT for one academic year, alternating sessions. Before each session, students were informed of therapy modality and rated their excitement using a Smiley Face Likert scale with associated text. To assess feasibility, the number and order of each session type, as well as student refusal rate, were investigated. To assess engagement, the therapist recorded time-on-task and number of times redirection was needed. Feasibility was reported as descriptive statistics, and Wilcoxon signed-rank tests were used to compare average scores on excitement and engagement between AT and standard PT.

**Results:**

60% of students (9/15) reaching the intended goal of 50% of therapy sessions as AT, as well as 60% (9/15) of students ‘roughly alternating’ (no more than three same session types in a row) their sessions throughout the duration of the study. The student refusal rate of AT was 0%. When using AT, students were 23% (*p*= <.0001) more excited, 4.4% (*p*=.004) more on task and needed 61% (*p*= <.0001) less refocusing compared to standard PT.

**Conclusion:**

Integrating AT alongside standard PT proved to be moderately feasible in a school setting. Additionally, students demonstrated increased excitement, increased time on tasks and decreased refocusing during a session using AT. A flexible-use approach will make AT a motivating addition to school-based therapy.

## Introduction

For many children diagnosed with a physical or learning disability, ongoing physical (PT) or occupational (OT) therapy is a part of life, often delivered in the school setting. About 15% of students in public schools receive some form of PT, OT or speech therapy to address diagnoses such as attention-deficit/hyperactivity disorder (ADHD), autism, developmental delay and global apraxia [1]. Participating in a regularly scheduled therapy intervention can be crucial for students’ development; research shows that individualized in-school therapy can improve physical functioning and activities of daily living as measured by the School Function Assessment [2]. The success of the intervention, however, is largely affected by the students’ active participation.

When students are more actively physically engaged, they tend to score higher on functional outcome assessments [2]. Conversely, therapists have observed various factors that decrease patient engagement and found that decreased engagement and involvement will likely limit a patient’s progress [3]. Despite the numerous strategies pediatric therapists utilize to keep students focused, keeping young children active and engaged during therapy can be challenging. The school environment, in particular, is full of opportunities for distraction and decreased engagement. Throughout a PT session, therapists frequently find themselves refocusing their students and redirecting them to the task at hand. School therapy sessions are limited in time and therefore time spent coaxing students to participate fully and redirecting after distractions steals time from active participation in therapeutic exercise.

Recent advancements in technology can offer solutions to decreased engagement and participation by expanding the options that physical therapists have at their disposal. Virtual (VR) and augmented (AR) reality platforms are being used in a variety of ways to enhance pediatric therapy, typically resulting in increased engagement [4,5]. By leveraging the nearly limitless possibilities of technology, the same therapeutic activities can be presented in new and different ways. The excitement associated with new technology, such as mobile exergaming, has led to increases in voluntary daily activity and step count in a school-based setting [6]. In addition to increasing general activity, virtual, augmented and mixed reality-based interventions have proved beneficial for ADHD symptoms [7,8], autism [9] and intellectual disabilities [10] in children, adolescents and young adults.

Adding new technology to a school-based therapy program has the potential to increase student engagement and therapy participation, however, there are still barriers to overcome. Many interactive technology systems are expensive, require specialized equipment, can be cumbersome to set up in different locations and are subject to infection control issues with wearable headsets or hand-held controls. In addition to hardware challenges, studies suggest difficulties in achieving long-term engagement with exergaming as an intervention [11]. Augment Therapy^®^ (AT) software was designed to address these challenges with an interactive therapy platform run on an iOS tablet where participants interact with the software *via* camera-enabled motion capture technology – providing a touch-free environment for the student [12]. Additionally, the software includes over 40 different therapy experiences to provide variety to keep users interested over time.

Despite the popularity of AR, VR and exergaming for health benefits [11], little is known about the implementation and usage of these types of technologies for long-term therapy in the school setting. Consequently, the purpose of this feasibility study was to determine if a therapist would be able to integrate exergaming technology into their current practice and to understand if participation with a new AR-based therapy technology could affect student engagement and motivation for PT in a school-based setting. By integrating AT technology in a real-world setting for an extended period of time, this study will help inform future technology implementations and design considerations within the physical therapy community. We hypothesize that the therapist will be able to utilize AT for an entire academic school year, provide approximately 50% of students’ therapy with AT and that students will have a low refusal rate when presented with AT for therapy. Additionally, we anticipate that AT will be at least as engaging as standard PT, measured by enthusiasm for the therapy session, time on task and number of redirections.

## Materials and methods

### Study design

This single cohort feasibility study compared two conditions and pursued a Hybrid Type 2 study approach. All study interventions and outcome measures were completed in the school setting during regularly scheduled PT sessions and measures were collected for each intervention session. Study duration spanned the entire 2022–2023 school year.

### Participants

A convenience sample of 15 students ages 6–8 years (11 males, 4 females) with an Individual Education Plan, receiving once-weekly PT participated in the study. The onsite physical therapist determined student eligibility by their ability to successfully interact with the software and if AT provided the exercises targeting the students’ individual therapeutic needs. Inclusion criteria: students with a diagnosed physical or learning disability, qualified to receive school physical therapy as part of their Individual Education Plan (determined by a multidisciplinary school team), had therapeutic needs that could be addressed with AT activities and could follow two-step directions. Exclusion criteria: students who were physically or cognitively unable to interact with the AT software. Study protocol was approved by University of Dayton’s Institutional Review Board (IRB No. FWA00015321, Ticket ID No. 19814422), the school’s principal and the regional therapy center. This study adhered to the principles according to the ‘Declaration of Helsinki’. Informed parental written consent was obtained for all students. See Table 1 for additional subject demographics.

**Table 1.** Subject population demographics.

DEMOGRAPHICS	n	% TOTAL
Age		
6	2	0.13
7	6	0.40
8	7	0.47
Grade		
K	2	0.13
1	3	0.20
2	9	0.60
3	1	0.07
Sex		
Female	4	0.27
Male	11	0.73
Ethnicity		
White	14	0.93
Hispanic	1	0.07
Diagnosis		
ADHD	4	0.27
Autism	1	0.07
Developmental delay	2	0.13
Seizure disorder	1	0.07
Global apraxia	1	0.07
Other diagnosis	6	0.40

*Note:* N: number of subjects; % Total: percentage of total subject population; ADHD: attention-deficit/hyperactivity disorder.

**Procedure** (section moved from after to before ‘Measures’ to provide better context to outcome measure explanation)

**Figure 1.**
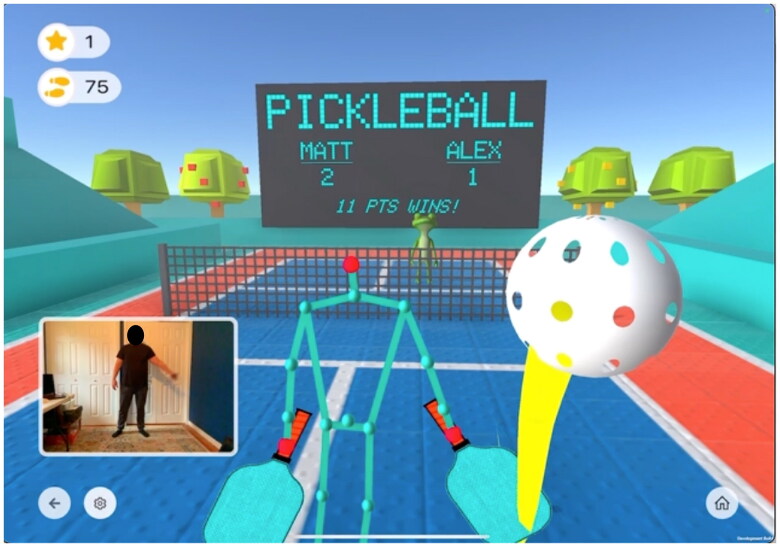
Example of an augment therapy game.

All students’ physical therapy goals included targeting strength, balance and coordination. Therefore, both standard therapy and AT focused on these areas for all students. Standard physical therapy included activities such as age-appropriate recreational games like t-ball, tennis, jumping rope and ball passing; body awareness activities including body rolling, balance beam and trampoline jumping; and calisthenics such as jumping jacks, sit-ups, push-ups and mountain climbers. Augment Therapy included AR games and exercises that incorporated standard physical therapy exercises in a simulated game environment where the student visualized themselves on the screen while completing the tasks (Figure 1). All therapy sessions were approximately 20 min in length.

The physical therapist provided either standard physical therapy or AT to students, with instructions to ‘roughly alternate’ session modalities (no more than three of the same session types in a row) and to provide half standard therapy and half AT to the best of their ability. Due to space and time availability, intervention location varied. Intervention locations included a gym, playground, hallway and classroom.

### Measures

Feasibility was assessed using measures of fidelity, reach and sustainability [13]. Fidelity was defined as the percentage of therapy sessions that were completed with AT (when a student completed an odd number of total sessions, the total percentage was considered 50% if the difference between the number of sessions modalities was only one). Reach was defined by student refusal rate of AT sessions. Sustainability was defined by the ability of the therapist to maintain a ‘roughly alternating’ schedule (no more than three of the same session types in a row) throughout the entire study duration and is represented as a percentage of students who met this criteria.

Intervention outcome measures included excitement for the upcoming therapy session, time on task and number of redirections. Excitement was defined by qualitative answers to the question ‘How excited are you for therapy today?’, with answer options: ‘very unexcited’, ‘a little unexcited’, ‘I don’t know’, ‘a little excited’, ‘very excited’ with an associated Smiley Faces Likert scale. Time on task was defined as percent of time a student spent in active therapy during each session, with answer options: ‘0–20%’, ‘21–40%’, ‘41–60%’, ‘61–80%’, ‘81–100%’. The number of redirections was defined as the number of instances where the therapist needed to refocus the student and direct their attention back to the therapy activity. The same therapist collected all data points throughout the study.

### Data analysis

Feasibility metrics were reported as descriptive statistics. Time on task and motivation metric outcomes were both converted to a 1–5 scale prior to analysis. Shapiro–Wilk tests determined non-normal distribution among the datasets. Therefore, Wilcoxon signed-rank tests were performed on all intervention outcome metrics comparing the AT and standard therapy conditions.

## Results

60% of students (9/15) reaching the intended goal of 50% of therapy sessions as AT, as well as 60% (9/15) of students ‘roughly alternating’ (no more than three same session types in a row) their sessions throughout the duration of the study. The student refusal rate of AT was 0%.

**Figure 2.**
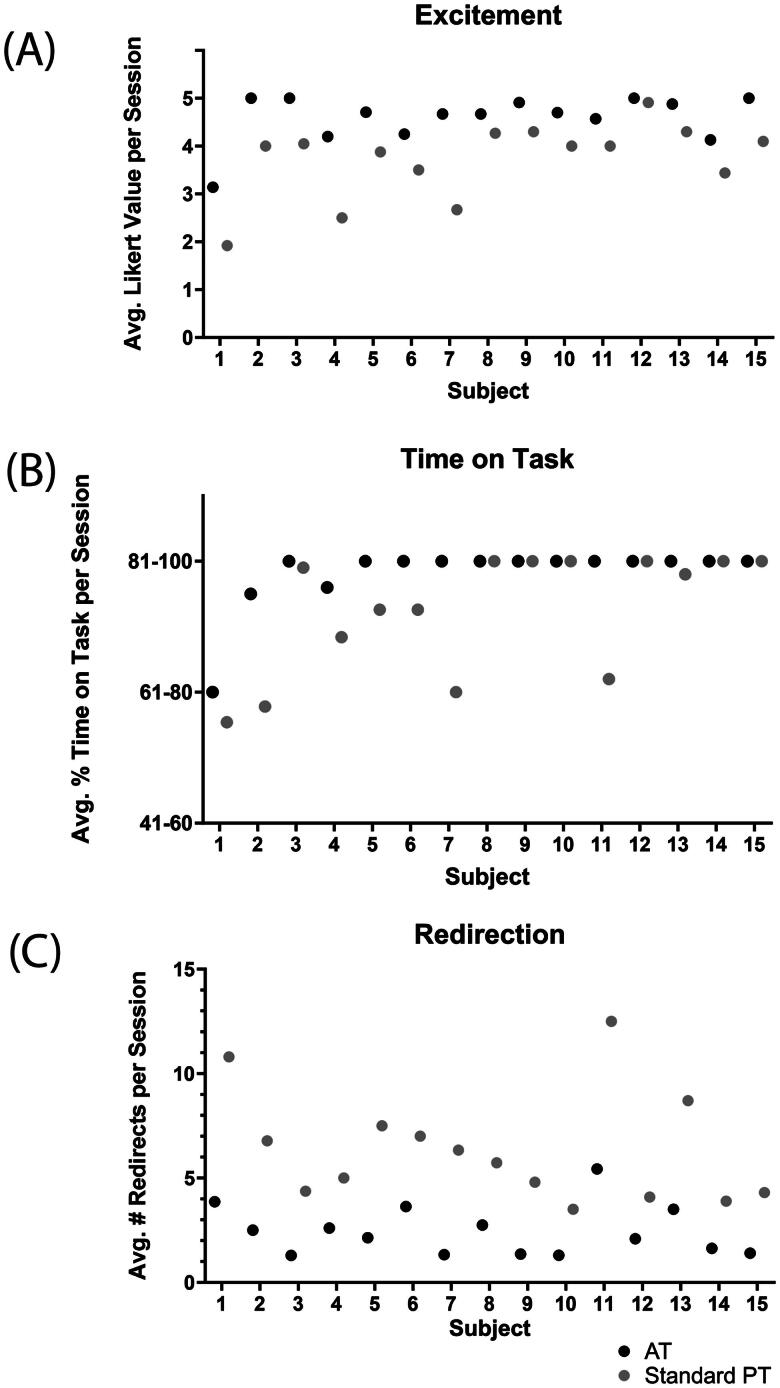
Mean scores for augment therapy (AT) and standard therapy (PT) for (A) excitement reported as average (avg.) Likert value per session, (B) time on task reported as average (avg.) percent (%) time on task per session and (C) redirection reported as average (avg.) number (#) of redirects per session.

Significant differences were found between standard therapy and AT on all intervention outcome metrics. When students participated in AT they were 23% more excited for their therapy session (*n* = 15, W= −120.0, *p*<.0001), had 4.4% greater time on task (*n* = 9, W= −45.0, *p*=.004) and needed 61% less redirection (*n* = 15, *W* = 120.0, *p*<.0001) compared to standard PT. Figure 2 displays each subject’s average score for each metric. Augment Therapy was integrated alongside traditional PT in the school setting with no student refusals for AT, however, the therapist was unable to strictly maintain the alternating schedule due to changing space availability and student schedules (i.e. sometimes the only space available at a particular time for a student did not yield itself to AT, such as outside at recess).

## Discussion

This study evaluated the feasibility of using AT software, a gamified, interactive therapy platform, as a part of school-based PT interventions in children with motor impairments. The results imply that the use of AT, which incorporates AR games and exercises, is not only a feasible addition to traditional PT, but may also improve focus and engagement, time on task and student excitement and motivation during PT sessions.

The feasibility outcome metrics suggested moderate success of in-school technology integration. Although only 60% of students met the goal of roughly alternating session types with 50% of the sessions including AT, valuable anecdotal evidence provided context to these results. According to the school therapist, factors related to therapy location often dictated the type of therapy offered. Some examples where the therapist could not use AT included not enough space for the minimum distance required between the device and the student, the ambient noise was too loud to hear the audio from the device and AT creating a potential distraction for other students in a shared therapy space. While these space constraints caused limitations, AT was also easy to transport and could be used in many other situations where a larger technology setup (such as a rolling cart with a television) would not be feasible, therefore illuminating a significant strength of this particular intervention. As suggested by Gefen et al. [14], gathering a holistic understanding of real-world functionality is crucial to a technology implementation strategy. These results can inform future design decisions as well as opportunities for further research.

In contrast to other exergaming interventions that have shown difficulties with long-term adoption [11], our results suggest sustainability using AT technology in a school setting. Exploratory analysis revealed that all subjects had at least 50% of their AT sessions occur in the second half of the school year, indicating that the software maintained its relevance and usefulness as a therapeutic tool over time. Furthermore, a 0% student refusal rate reinforced the longevity of the technology as a desirable form of therapy.

The results of this study associated with the intervention are consistent with similar studies that utilize gaming and AR/VR technology for pediatric physical therapy interventions. The addition of gamified technology to physical interventions has resulted in increased exercise participation in an obese population [15], increased excitement of a child who had previously refused physical therapy [4] and improved motivation in gait rehabilitation with the addition of a video game component [5]. The results from this study add to these previous findings with a similar increase in excitement when gamified technology is introduced. Additionally, in this study increased excitement is accompanied by increased focus and participation and is consistent with other studies that found improved participation [16] and longer participation [4] with exergaming. Furthermore, the clinical implications of increased excitement during an intervention are improved outcomes due to increased adherence, participation intensity and intervention time metrics [17].

The valuable information gained from this study supports the use of AT as an adjunct therapy tool to use in the school setting and provides additional insight into the situational considerations a therapist must make when working with students day to day. Although using a therapy tool like AT is feasible and effective, further design and functionality developments informed by the results of this study will improve the software’s usability and potentially its effectiveness.

## Strengths

This study demonstrated sustained engagement with a novel AR-based therapy tool used in the school setting for an entire school year. Additionally, when compared to standard PT, students were more engaged and motivated on average.

## Limitations

One therapist conducted all therapy sessions and collected all outcome data for both therapy modalities, which introduced a risk of bias. This also eliminated the possibility to test for staff adoption as a feasibility outcome metric. Additionally, the current sample size was small and did not include a wide range of ages. Future studies should include multiple therapists/providers to allow for adoption testing and to broaden the sample population.

## Implications

Our results support therapists integrating exergaming technologies alongside standard therapy in the school setting as both a feasible and effectively engaging therapy tool to provide greater variety in therapeutic exercises. Educators may also utilize this technology for ‘activity breaks’ to incorporate focused physical activity in the classroom; further research is warranted in this area. This study also demonstrates the importance of investigating new therapeutic technologies in real-world settings to understand and develop the most effective implementation methods.

## Data Availability

The data that support the findings of this study are available from the corresponding author, [EB], upon reasonable request.

## References

[1] National Center for Education Statistics. COE - students with disabilities [Internet]. 2023 May [cited 2024 Apr 10]. Available from: https://nces.ed.gov/programs/coe/indicator/cgg/students-with-disabilities

[2] Mccoy SW, Effgen SK, Chiarello LA, et al. School‐based physical therapy services and student ­functional ­performance at school. Develop Med Child Neuro. 2018;60(11):1140–1148. doi: 10.1111/dmcn.13748. 29603734

[3] Lequerica AH, Donnell CS, Tate DG. Patient engagement in rehabilitation therapy: physical and occupational therapist impressions. Disabil Rehabil. 2009;31(9):753–760. doi: 10.1080/09638280802309095. 19034722

[4] Hemphill S, Nguyen A, Kwong J, et al. Virtual reality facilitates engagement in physical therapy in the pediatric CVICU. Pediatr Phys Ther. 2021;33(1):E7–E9. doi: 10.1097/PEP.0000000000000769. 33337780

[5] Sanpablo AIP, Armenta-García JA, Muñiz AF, et al. Integration of persuasive elements into exergames: application in the development of a novel gait rehabilitation system for children with musculoskeletal conditions. J Biomed Inform. 2022;132:104130. doi: 10.1016/j.jbi.2022.104130. 35820597

[6] Garde A, Umedaly A, Abulnaga SM, et al. Evaluation of a novel mobile exergame in a school-based environment. Cyberpsychol Behav Soc Netw. 2016;19(3):186–192. doi: 10.1089/cyber.2015.0281. 26882222

[7] Benzing V, Schmidt M. The effect of exergaming on executive functions in children with ADHD: a randomized clinical trial. Scand J Med Sci Sports. 2019;29(8):1243–1253. doi: 10.1111/sms.13446. 31050851

[8] Goharinejad S, Goharinejad S, Hajesmaeel-Gohari S, et al. The usefulness of virtual, augmented, and mixed reality technologies in the diagnosis and treatment of attention deficit hyperactivity disorder in children: an overview of relevant studies. BMC Psychiatry. 2022;22(1):4. doi: 10.1186/s12888-021-03632-1. 34983446 PMC8728980

[9] Lima JL, Axt G, Teixeira DS, et al. Exergames for children and adolescents with autism spectrum disorder: an overview. Clin Pract Epidemiol Ment Health. 2020;16(1):1–6. doi: 10.2174/1745017902016010001. 32508964 PMC7254818

[10] Ryuh YJ, Chen C-CJ, Pan Z, et al. Promoting physical activity through exergaming in young adults with intellectual disabilities: a pilot study. Int J Dev Disabil. 2022;68(2):227–233. doi: 10.1080/20473869.2019.1605771. 35309694 PMC8928788

[11] Benzing V, Schmidt M. Exergaming for children and adolescents: strengths, weaknesses, opportunities and threats. J Clin Med. 2018;7(11):422. doi: 10.3390/jcm7110422. 30413016 PMC6262613

[12] Augment Therapy [Internet]. Augment therapy | immersive augmented reality exercise apps for clinic and home [cited 2024 Apr 10]. Available from: https://www.augmenttherapy.com

[13] Pearson N, Naylor PJ, Ashe MC, et al. Guidance for conducting feasibility and pilot studies for implementation trials. Pilot Feasibility Stud. 2020;6(1):167. doi: 10.1186/s40814-020-00634-w. 33292770 PMC7603668

[14] Gefen N, Mazer B, Krasovsky T, et al. Novel rehabilitation technologies in pediatric rehabilitation: knowledge towards translation. Disabil Rehabil Assist Technol. 2024:1–10. doi: 10.1080/17483107.2024.2445017. 39727293

[15] González-González CS, Gómez Del Río N, Toledo-Delgado PA, et al. Active game-based solutions for the treatment of childhood obesity. Sensors. 2021;21(4):1266. doi: 10.3390/s21041266. 33578854 PMC7916583

[16] Hall L, Hume C, Tazzyman S. Five degrees of happiness: effective smiley face Likert scales for evaluating with children. 2016:311–321. doi: 10.1145/2930674.2930719.

[17] Fragala-Pinkham MA, Haley SM, Rabin J, et al. A fitness program for children with disabilities. Phys Ther. 2005;85(11):1182–1200. doi: 10.1093/ptj/85.11.1182. 16253047

